# Contraceptive knowledge, perceptions, and concerns among men in Uganda

**DOI:** 10.1186/s12889-017-4815-5

**Published:** 2017-10-10

**Authors:** Nityanjali Thummalachetty, Sanyukta Mathur, Margo Mullinax, Kelsea DeCosta, Neema Nakyanjo, Tom Lutalo, Heena Brahmbhatt, John S. Santelli

**Affiliations:** 1 Heilbrunn Department of Population and Family Health, Columbia University Mailman School of Public Health, 60 Haven Ave., B-2, New York, NY 10032 USA; 2 HIV Center for Clinical and Behavioral Studies, Columbia University and New York State Psychiatric Institute, New York, NY USA; 3 Rakai Health Sciences Program, Kalisizo, Uganda; 4 Department of Population, Family and Reproductive Health, John Hopkins Bloomberg School of Public Health, Baltimore, MD USA

**Keywords:** Men, Contraceptive knowledge, Contraceptive side effects, Interpersonal, Contraceptive decision-making

## Abstract

**Background:**

Low contraceptive uptake and high unmet need for contraception remain significant issues in Uganda compared to neighboring countries such as Kenya, Ethiopia, and Rwanda. Although prior research on contraceptive uptake has indicated that male partners strongly influence women’s decisions around contraceptive use, there is limited in-depth qualitative research on knowledge and concerns regarding modern contraceptive methods among Ugandan men.

**Methods:**

Using in-depth interviews (*N* = 41), this qualitative study investigated major sources of knowledge about contraception and perceptions of contraceptive side effects among married Ugandan men. RESULTS: Men primarily reported knowledge of contraceptives based on partner’s experience of side effects, partner’s knowledge from health providers and mass media campaigns, and partner’s knowledge from her peers. Men were less likely to report contraceptive knowledge from health care providers, mass media campaigns, or peers. Men’s concerns about various contraceptive methods were broadly associated with failure of the method to work properly, adverse health effects on women, and severe adverse health effects on children. Own or partner’s human immunodeficiency virus (HIV) status did not impact on contraceptive knowledge.

**Conclusions:**

Overall, we found limited accurate knowledge about contraceptive methods among men in Uganda. Moreover, fears about the side effects of modern contraceptive methods appeared to be common among men. Family planning services in Uganda could be significantly strengthened by renewed efforts to focus on men’s knowledge, fears, and misconceptions.

## Background

Despite renewed emphasis on family planning services in Uganda through global partnerships such as Family Planning 2020 and local efforts committed to promoting gender inclusiveness by organizations such as Reproductive Health Uganda, low contraceptive uptake and high unmet need for contraception remain significant issues in Uganda compared to neighboring countries such as Kenya, Ethiopia, and Rwanda [1–3]. In a 2011 Demographic and Health Survey (DHS), about one-third of currently married women had an unmet need for family planning [4]. Among both women and men, knowledge of at least one contraceptive method was universal. However, over half of women report a lack comprehensive knowledge of these methods, including awareness of side effects, and only 59% were given information about other methods by a health worker [4]. There is no information on men’s comprehensive knowledge about contraceptive methods in this survey. Nationally, a majority of both men and women receive family planning messaging from the radio and more men than women report seeing messages on television or in print media (25 and 15% respectively for each medium) [4]. Overall, there is a clear need for continued efforts to promote contraceptive knowledge and use in Uganda.

One approach for increasing uptake of contraceptives among women has been the inclusion of men in family planning programming. The 1994 International Conference on Population and Development in Cairo reinforced the need to engage men in family planning interventions and acknowledge their role in reproductive health [5]. Research suggests that although contraceptive decision-making often occurs at the couple-level, men have considerable influence on these decisions. For example, research in Ethiopia demonstrates that more than half of a sample of married men reported joint decision-making on when to have another child and when to stop child bearing, and the majority of respondents reported joint decision-making about the method of contraceptive to use [6]. Men’s key role in family planning decision-making is also supported by research in other parts of Africa [7]. Furthermore, men’s knowledge of and involvement in family planning is a significant predictor of uptake and use of contraception within couples [5, 8, 9]. Moreover, the Malawi Male Motivator intervention, a peer-delivered intervention targeting men to increase couples’ contraceptive uptake, revealed significant increase in contraceptive use post-intervention [10]. Additionally, ease and frequency of communication about family planning within couples significantly predicts contraceptive uptake, which is an indication that men influence contraceptive use and choice and use of contraception is derived from joint decision-making [10].

Studies have found that often, ‘husband/partner is opposed’ is listed as one of the primary reasons non-users report for not using contraception, and women’s lower uptake and discontinuation of contraception is strongly influenced by their male partner’s lack of proper knowledge about and resistance to the use of family planning methods [11–15]. Additionally, women in Zambia have reported covert contraceptive use due to husband disapproval of contraceptive use [16]. Even when controlling for women’s own fertility desires, men’s desires can be both a perceived and actual barrier to family planning uptake [17].

Given the importance of men in family planning, some research has examined men’s contraceptive knowledge (for example, Oyediran, Ishola, & Feyisetan, 2002, UBOS and ICF International Inc., 2012); however, many of these studies generally use a simplistic measure of contraceptive awareness (as opposed to knowledge about contraception) [4, 18]. For example, research with ever-married men in Nigeria demonstrated high levels of contraceptive knowledge combined with high levels of usage [18]. Yet, researchers in this study only assessed contraceptive knowledge using “having heard of” a particular contraceptive method as an overall measure of knowledge about modern contraception. Moreover, there were no measures to validate men’s accuracy of knowledge. Similarly, the most recent DHS Report in Uganda reported high awareness of modern contraceptive methods (~90% or higher) among men without an assessment of how the methods are used or their potential side effects [4]. In-depth qualitative research exploring men’s knowledge about modern contraceptive methods remains a nascent field [19]. In Uganda, there is an emerging body of qualitative literature that focuses on factors influencing men’s perceptions of contraceptive use [11, 12, 20]. However, there is limited research on men’s sources of knowledge regarding modern contraception. As a result, there is a need for in-depth qualitative exploration of men’s knowledge and perceptions of modern contraception, including sources of knowledge, how these methods are used, and knowledge regarding potential side effects and benefits of particular methods.

This paper aims to fill gaps in knowledge and capture the circumstances and sources from which men in Uganda gain knowledge about contraception, and their subsequent perceptions of different contraceptive methods. This paper serves to increase understanding of men’s interaction with family planning messaging, where findings may be used to create targeted messages for men around particular contraceptive methods.

## Methods

### Prevention and planning linkages project study overview and design

Data for this paper derive from the Prevention and Planning Linkages project, which uses mixed-methods to explore the intersection of HIV risk and fertility desires and outcomes among men and women in the southwest district of Rakai, Uganda. In the qualitative arm of this study we focused on examining relationship dynamics and reproductive desires and behaviors within the context of HIV. We conducted face-to-face interviews in a private space in the respondent’s home or a location of the respondent’s choosing near their home. Interviews were conducted by trained Ugandan qualitative researchers. Men were interviewed by male interviewers who speak the local languge and are familiar with local customs to facilitatie quick rapport during interviews. We collected data from 41 married (consensual or formal marriages) couples selected from the Rakai Community Cohort Study (RCCS) in southwestern Uganda; the RCCS has been described in detail elsewhere [21]. As part of this cohort, participants have access to a range of reproductive health services including family planning, STI treatment, HIV counseling and testing, and HIV care [22]. The RCCS cohort includes participants from three types of communities, classified by the study teams as rural agrarian, trading communities (i.e. peri-urban), and fishing communities along the shore of Lake Victoria in Uganda [23]. It is worth noting that fishing communities have higher rates of HIV prevalence than the peri-urban trading communities and rural agrarian communities [23]. Couples were selected on the basis of number of children female partners within couples had (categorized as no children, one to three children, and more than three children) and the couple’s HIV status (sero-concordance and sero-discordance). For this secondary data analysis of our qualitative data, we used data from the 41 men aged 20 to 50 years.

### Measures and procedures

During interviews, all respondents regardless of HIV status were asked about their fertility desires, communication with their partners, experiences with HIV testing and treatment, and knowledge and use of contraception. The following are some examples of questions in the interview guide related to contraceptives: “When you first got married to [your] partner, did you use any birth control methods?”, “What concerns did you have about using birth control?”, “Were there times when you wanted to use a method of birth control but you were not able to?”, and “Did you ever receive any advice from a health care provider about [a] birth control method?” Responses about contraception in the larger interview guide elicited themes about sources of contraceptive knowledge and perceptions about contraceptives. This paper focuses on those emergent themes.

Male interviewers conducted in-depth semi-structured interviews with all consenting respondents. All interviews lasted between an hour and 1.5 h, and were conducted in a private setting, out of earshot of acquaintances and family members. Respondents were compensated ~$3 US for their time, per Ugandan ethical guidelines. The interviews were conducted in Luganda (the local language) and audio recorded, then translated and transcribed in English. Interviewers also wrote field notes, including summaries of their reflections and observations immediately following each interview, all of which were included in the body of qualitative data that was collected and analyzed.

Data collection was approved by IRBs at Columbia University, by the Research and Ethics Committee at Uganda Virus Research Institute, and by the National Council for Science and Technology in Uganda.

### Data analyses

We focus this analysis specifically on themes related to contraception such as knowledge, points of access, and experience using contraception. We used NVivo 10 software for qualitative data management and analysis. We used thematic analysis to identify, analyze, and report patterns or themes from the data [24, 25]. This method of analysis involved close reviews of the transcripts and field notes to create an initial set of themes reflecting the original domains of the interview guide. Interview and field notes data were then broadly coded by the first author; we then identified a preliminary set of sub-codes based on repetitive patterns or meaning in the data that provided insight into the research aims of this study [25]. The first author further examined the preliminary set of coded data for relationships and intersections. We used broad coding for relevant text to retain pertinent context in the interview and field notes data. The first two authors independently analyzed the coded data and then discussed in detail areas of consensus and disagreements.

## Results

### Sample descriptive data

Our sample of 41 respondents included men ranging from 20 to 50 years of age, with a median age of 34 years. Approximately 41% of respondents resided in peri-urban areas, followed by 32% in fishing communities and 27% in rural areas. Over 46% of respondents had four or more children, with the median number of children being 4 and ranging from none to 17. Approximately 59% of men reported not using any form of contraception, 22% reported using condoms, and 19% reported that their partner used some form of contraception (with Depo-Provera being the most commonly used method among partners). A little over half the respondents in our study (54%) were HIV negative and 46% were HIV positive. Sixty-three percent of respondents were in serodiscordant and 37% in seroconcordant relationships. For more demographic information detailing education level and occupation of our study respondents, please see Table 1. The category “other” under occupation in Table 1 includes occupations such as teachers, policemen, and boat managers.

**Table 1:** Demographic data and other characteristics (*N* = 41)

	Median	Range
Age	34	20–50
Number of Children	4	0–17
	Percentage (%)	
Education Level		
No Formal Education	2	
Some Primary Education	64	
Some Secondary Education	32	
Some Tertiary Education	2	
Residential Area		
Rural	27	
Peri-urban	41	
Fishing Communities	32	
Occupation		
Agriculture	15	
Fishing	17	
Traders/Shopkeepers	14	
Transportation	12	
Construction	10	
Other	32	
Current Contraceptive Use		
Condom	22	
Partner’s Contraceptive Use	19	
No Contraceptive Use	59	
HIV Status		
Negative	54	
Positive	46	
Couple-level HIV status		
Seroconcordant	37	
Serodiscordant	63	

Due to our small sample size, we did not find differences in men’s knowledge, perceptions, or concerns about contraceptive methods or their side effects based on their age, HIV status, their HIV seroconcordant or serodiscordant partnerships, or their residence in trading, rural, or fishing (typically HIV high-risk) communities; therefore, we present pervasive and emergent themes across respondents in this study.

#### Results 1: Men’s sources of information

**Fig. 1:**
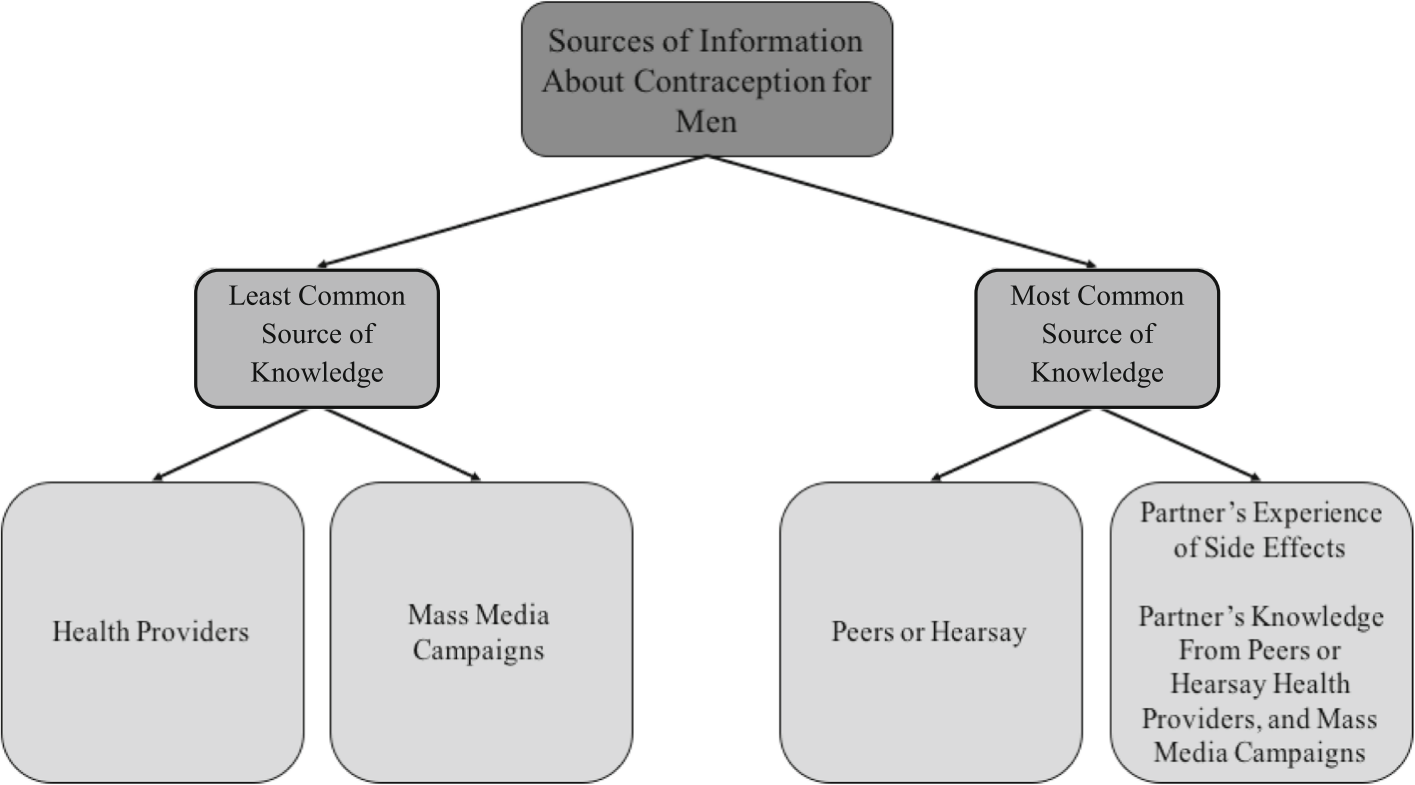
Sources of information about contraception for men

Interview data from our study indicated that men received knowledge about contraception from a number of sources (see Fig. 1). Men reported having received information about contraception from their peers, health care providers, or mass media campaigns. Second-hand information from partners came from a combination of the following four major sources: 1. Partner’s experiences of side effects, 2. Partner’s knowledge from peers or hearsay, 3. Partner’s knowledge from health providers, or 4. Partner’s knowledge from mass media campaigns. Men in this study acquired most information about contraception from their partners, their own peers, or hearsay. Few men reported having received information about contraceptives from mass media campaigns or health providers. Much of men’s reported knowledge about contraception in this study indicated ineffectiveness of methods to prevent pregnancies and adverse health effects on women and future children.

#### Results 2: Beliefs about contraceptives among men

Our data on men’s beliefs about contraception revealed three major themes: 1. Failure of the method to work properly and prevent pregnancy; 2. Adverse health effects on women; and 3. Adverse health effects on children.

### Failure of the method to work properly and prevent pregnancy

A common theme in this study was concerns about contraceptive methods such as the IUD or condoms being displaced (or moving) within the body, and subsequently failing to prevent pregnancy. Men reported their own as well as their partners’ fears about the IUD getting displaced in the woman’s body, resulting in an unplanned pregnancy. For instance, one 34 year old respondent with more than four children explained that this wife had expressed being uneasy with the IUD. He stated, *“…she complained that she felt uneasy with it. She was told that the [IUD] could easily get lose and becomes pregnant…that the [IUD] can loosen and drops without her knowledge.”* Similarly, another 31 year old man with three children, elucidated that he and his female partner had been concerned that the IUD was harmful because they had heard that it could get displaced in the body. He reported, *“Some people say [IUDs] get displaced in the body though some refute this belief. Others say it remains stationed in one place, how does it stop pregnancy then.”* In addition to voicing concerns about the IUD being displaced in women’s bodies, men also expressed their own and their partners’ concerns about condom use. A 27 year old man who have one child in our study explained, *“I was told that if you fail to use it well, that condoms can easily get stuck in the woman. That made me get worried about it”.* Some men also reported their female partners’ refusal to use condoms due to fears of having condoms stuck in their bodies. One 33 year old man with two children explained that his partner feared death due to having a condom stuck inside her body. He stated, *“I suggested to her that we use condoms but she refused and told me that she heard about a woman who had a condom stuck (remained) in her vagina and she died. She refused to use a condom.”* Men’s perceptions of contraceptive methods such as the IUD and condoms as being unreliable were informed by men’s own beliefs as well as their partners’ beliefs to some degree. Moreover, men reported doubts in the actual functioning of the methods in the body, leading to concerns about failure of the methods in preventing pregnancies.

### Adverse health effects on women

Men in this study reported a variety of potential side effects resulting from contraceptive methods commonly used in Uganda. Table 2 provides a detailed list of side effects reported by respondents by the contraceptive type. It is worth noting that hormonal methods such as Depo-Provera, contraceptive pills, and the IUD were associated with the most extreme side effects in women. Moreover, men appeared to have the most concerns about side effects associated with contraceptive pills.

**Table 2:** Respondent perspectives on side effects of particular contraceptive methods

Contraceptive Method	Reported Adverse Side Effects for Women and Children (*denotes adverse effects associated with children)
Depo-Provera	• Infertility
	• Weight gain
	• Burning of the reproductive eggs
Contraceptive Pills	• Infertility
	• Development of fibroids
	• Weight loss
	• Weakness
	• Loss of sexual fluids/dryness during intercourse
	• Burning of reproductive eggs
	• Prolonged and excessive menstruation
	• Uterine cancer
	• Spoiling of the blood
IUD	• Infertility
	• Displacement of the IUD and unplanned pregnancy
	• Problems during pregnancy and birth
	• Hypertension
	• Weakened limbs in women
Condoms	• Can get stuck in women’s bodies (could cause death)
	• Infections in women’s sexual organs
	• Sexual weakness among men due to tightness of condom
Norplant	• Infertility
	• Spoiling of the blood

#### Concerns about reproductive morbidities

Men’s concerns about adverse side effects of contraceptives often included those that are commonly associated with contraceptive use in the US and Europe such as prolonged menstruation, changes in weight, or changes in vaginal lubrication during intercourse [26–28]. Men also reported concerns about more severe side effects such as hypertension and weakness in the limbs, the development of infections or fibroids in women’s reproductive organs, and uterine cancer. For example, one 50 year old respondent with fifteen children (five with his current partner) explained that his female partner was afraid to use the IUD method because she had heard that it weakened the limbs, such as the hands, and could cause hypertension. Respondents also expressed their beliefs about condoms causing infections in women’s bodies. As one 40 year old respondent with six children stated, *“They tell us condoms can cause something in the woman’s sexual organ… she can develop a rash inside the sexual organ”*. Another 30 year old respondent with three children, clarified that he did not want his wife to use contraceptive pills (“pill plan”) because they caused fibroids in women. Oral contraceptive pills appeared to be particularly troublesome for many respondents, as is evident in Table 2. One 31 year old respondent with three children reported that the contraceptive pill use could cause uterine cancer in a woman’s body: *“They say pills are not safe, they cause cancer of the uterus. When you take pills, they cause a spot where they rest in the body and later cause an infection.”* Although men reported specific concerns about individual methods of birth control, overarching concerns about adverse side effects of contraceptive methods on female partners was a common theme among men’s discussions around use of family planning methods.

#### Fear of infertility

Respondents in our study commonly expressed fear of in fertility from contraceptive use among their female partners. In fact, as is evident in Table 2, infertility was associated with all methods used by women. As one 36 year old respondent with seven children explained, *“Some contraceptives “lock” the uterus and you may permanently stop to have children anymore. You may want to produce more children and fail to have them anymore.”* When asked about potential barriers to using birth control, another 20 year old respondent who did not have any children yet expressed needing more education about the IUD and asked the interviewer for further clarification about the method:

*Some women say that the coil affects their health. It makes them sick because their bodies [health] is not used to the coil. That is why I need education about it…There is something I need to know about the coil. Can a woman conceive if she wants to have a child if it (coil) is removed? I need to know this.* (20 year old male respondent)

Some study respondents also indicated that contraception such as Depo-Provera and pills caused burning of the ova in women, therefore resulting in infertility. A 29 year old respondent with two children stated, *“Some birth control methods destroy reproductive eggs and others make women sick. The injection makes women too fat and pills burn reproductive eggs.”* Another 21 year old with one child respondent whose wife was not using any contraceptive method explained, *“I hear that family planning injections burn down female reproductive eggs. If a woman has few potential eggs and she takes this injection, she might end up not producing children.”* Fear of infertility among women was a commonly cited barrier for using contraceptive methods by men. Moreover, phrases such as “locking of the uterus” or “burning of the eggs” were commonly used among respondents to express understandings of infertility as a side effect associated with contraceptive use. Additionally, some men, like in the example above, were interested in actively seeking more knowledge about possible side effects associated with particular methods.

#### Adverse health effects on children

In addition to side effects from contraception affecting female partners’ health, some men in our study also expressed concerns about negative health outcomes among future children. Fetal abnormalities resulting from contraceptive use were also a major cause of concern for some men in this study. Weak or deformed babies and problems during pregnancy were associated with improper use of contraceptive methods or “spoiled blood” (typically referring to contaminated blood caused by the use of hormonal contraception) in the mother’s body. One 30 year old respondent with 3 children who worried about how contraceptives might impact his wife’s health also expressed concerns about the improper use of Depo-Provera: *“If you fail to use injections properly you produce babies with disabilities…you might produce a baby with weak limbs or deformed legs.”* He further explicated, *“She has things in her body that are foreign, for example the IUD. It can cause trouble to her during birth…It can cause problems during pregnancy and to the baby.”* Additionally, the perception of spoiled blood due to contraceptives in the mother’s body was linked to infertility as well as weak babies. A 34 year old man with 4 children who had doubted the effectiveness of IUDs was also skeptical of long-acting injectable contraceptives. He explained that he and his female partner had not wanted to use contraceptives such as Norplant that “get directly into blood”: *“We have been told that pills spoil the blood and you can have a weak baby.”* This respondent further emphasized his concerns about the effects of prolonged use of contraception on fertility and adverse health outcomes among future children. He explicated,

*They say when you use such methods, the numbers of years you have used them are the years you remain without producing a child in case you stopped it and you want to get another child. For example if she uses it for 5 years she will take other 5 years before producing anoth er child when she stops using it. Some time you produce children who are mentally retarded, another thing the child may fall sick now and then. This is because the contraceptive method had already damaged the blood for long*. (34 year old male respondent)

In addition to side effects from contraception affecting female partners’ health, some men in our study also expressed concerns about negative health outcomes among future children. Misuse or prolonged use of hormonal methods such as Depo-Provera, the IUD, and Norplant were believed to result in fetal abnormalities among children.

## Discussion

In this study, we found that men received information about family planning from a wide variety of sources: their peers, health providers, or mass media campaigns, and their partners. Contraceptive information from peers and female partners appeared to be most often cited among men. Knowledge received from health providers or mass media campaigns were less commonly reported source of information among men, perhaps indicating that family planning programs are experiencing challenges with targeting men. Therefore, our study findings highlight an important gap in family planning messaging that could be filled by mass media and health care providers in Uganda. Moreover, our findings highlight that it is a fallacy to assume that men have high knowledge about contraception based on commonly used “ever heard of” measures in research or that men do not have strong opinions or ideas about contraceptive methods. Previous research on reproductive health and family planning, suggests that public health messages need to pay attention to belief systems, knowledge about anatomy and the body, and an understanding the mechanisms of (real or perceived) side effects of contraceptive methods [29–31]. Our study emphasizes the necessity of providing men with accurate contraceptive knowledge and basic and appropriate information about reproductive physiology, while understanding existent social and cultural belief systems.

We also found that misconceptions were very common and extensive among men. Other recent research on men’s knowledge about contraception in Uganda supports these findings. In a study conducted by Nalwadda et al. [12] exploring reasons for low contraceptive use among Ugandan youth, men expressed the fear of cancer, fibroids, reproductive morbidities, and infertility among their female partners caused by contraceptive pills, condoms, and the IUD. In another qualitative study by Hyttel et al. [11] investigating perspectives of injectable hormonal contraceptives in Uganda, men indicated concerns regarding excessive bleeding, menstrual irregularities, and lowered sexual desire among women. Similarly, these researchers report that men’s accounts of these side effects were informed by their partners’ own experiences of side effects and/or hearsay among community members about the method. Moreover, previous research from the RCCS community demonstrates the high desirability for and importance of childbearing among men in Uganda [32]. It is possible to speculate, based on findings from the present study, that men’s strong desire for children in combination with their misconceptions and concerns about contraception may negatively impact their support of partners’ use of contraception in Uganda.

We also expected that men in our study who were HIV positive or those in serodiscordant relationships would be particularly engaged with some type of health institution or facility that would be providing them with crucial contraceptive counseling and messaging. Yet, we were unable to identify discernable patterns of how individual or couple-level HIV status influenced men’s in-depth knowledge about contraception. We speculate that this may be a result of our small sample size and/or programmatic challenges that limit in-depth contraceptive knowledge from reaching Ugandan men.

Although the present study did not specifically explore men’s influence on women’s contraceptive uptake, research in Uganda suggests that gender inequities within relationships may result in limited decision-making power and negotiation among women whose male partners do not approve of contraceptive use [12, 33]. Moreover, women’s decisions to use contraceptives given actual or perceived side effects among men may cause sexual and domestic conflicts within couples, and men have considerable influence on contraceptive discontinuation among women [11, 12, 33]. There is a need for further in-depth research on men’s perceptions of and concerns with modern contraceptive methods and better ways to reach men with relevant information in order to more effectively address potential barriers to family planning uptake.

Our research elucidates some important barriers to contraceptive use among men, and subsequently women, in Uganda. There is a large body of research globally that provides invaluable insight into how gendered power dynamics and couple-level communication and negotiation regarding family planning influences women’s uptake of contraceptive use [11, 12, 34–38]. Our research adds to literature and highlights that, in addition to commonly cited gender inequities and power dynamics that strongly impact women’s contraceptive use, men’s concerns about adverse health outcomes among women and children pose an important barrier to family planning uptake that must be further studied. A more nuanced understanding of specific barriers among men related to accurate knowledge about contraceptives and the female reproductive biology would be critical for enabling program planning and policies aimed to improve contraceptive uptake among both men and women. Such programs and policies would likely facilitate meaningful avenues for intervention and change in contraceptive knowledge and uptake among men and women in settings with high levels of fertility and unmet need for contraception.

### Limitations

We found that men received information from a large range of sources, but we were unable to delineate the relative importance and specificity of one source over another. Additionally, we did not ask men what sources of information about contraception and reproductive health they would prefer – this may be an important question to explore as it may highlight why current program efforts often do not appear to result in accurate and comprehensive knowledge among men. During interviews, men frequently referred to sources of information as “they”, but it was unclear who specifically men were referring to – community members, peers, or health care providers. This limitation is particularly crucial and must be clarified in further studies as it may indicate a gap in communication from health care providers who are not articulating side effects of modern contraception clearly to their patients. In this analysis, we were unable to discern any substantial differences by individual or couple-level HIV status or by other demographic characteristics (i.e. area of residence, parity, age) in men’s knowledge, perceptions, or concerns about contraceptive methods or their side effects. However, these associations may have been difficult to detect due to our limited sample size and warrant further attention. This is particularly salient for couples that are serodiscordant since the assumption may be that they are receiving some type of contraceptive counseling and health information (especially to do with condom use) that would lower the risk of transmission between partners. We also did not investigate if men shared the same views as their partners regarding contraceptives – this may be an important theme to investigate in subsequent analysis. Finally, we were not able to demonstrate links between knowledge and perceptions of contraception and actual current contraceptive use given limitations in our data.

## Conclusion

Our study findings demonstrate that while men were engaged in conversations around contraception, they were notably misinformed about methods and side effects. Our data suggests that decisions around the use or disuse of particular contraceptive methods were often informed by efforts to protect female partners and, in some cases future children, from adverse side effects or birth defects. We speculate that this type of misinformation is at least partly a result of a dearth in family planning messaging focused on men combined with men’s erroneous understandings of women’s reproductive anatomy and health. Given the considerable body of existing literature on men’s influence on women’s uptake of contraception, and low contraceptive uptake among women despite renewed efforts in family planning agendas, there is an imperative need to better understand and address men’s knowledge and concerns about contraception as well female anatomy and reproductive physiology. Additionally, a close examination of how different sources of information influence men’s knowledge about family planning methods is necessary. Supporting strategies for national-level campaigns, as well as community-level mobilization targeting men are needed to subsequently enable avenues for higher demand, uptake, and sustained use of contraceptives in Uganda.

## Abbreviations

DHS: Demographic Health Survey
HIV: Human Immunodeficiency Virus
RCCS: Rakai Community Cohort Study
RHSP: Rakai Health Sciences Program
